# Supplementary material for: Cystatin C loaded in brain-derived extracellular vesicles rescues synapses after ischemic insult in vitro and in vivo
Source: Cell Mol Life Sci. 2024 May 20;81(1):224. doi: 10.1007/s00018-024-05266-4 (PMC11106054; doi:10.1007/s00018-024-05266-4)
Supplement: Supplementary file 1 — Supplementary file1 (DOCX 1091 KB) [file 18_2024_5266_MOESM1_ESM.docx]

**SUPPLEMENTAL FIGURES**

**Supp. Fig. 1.**

**
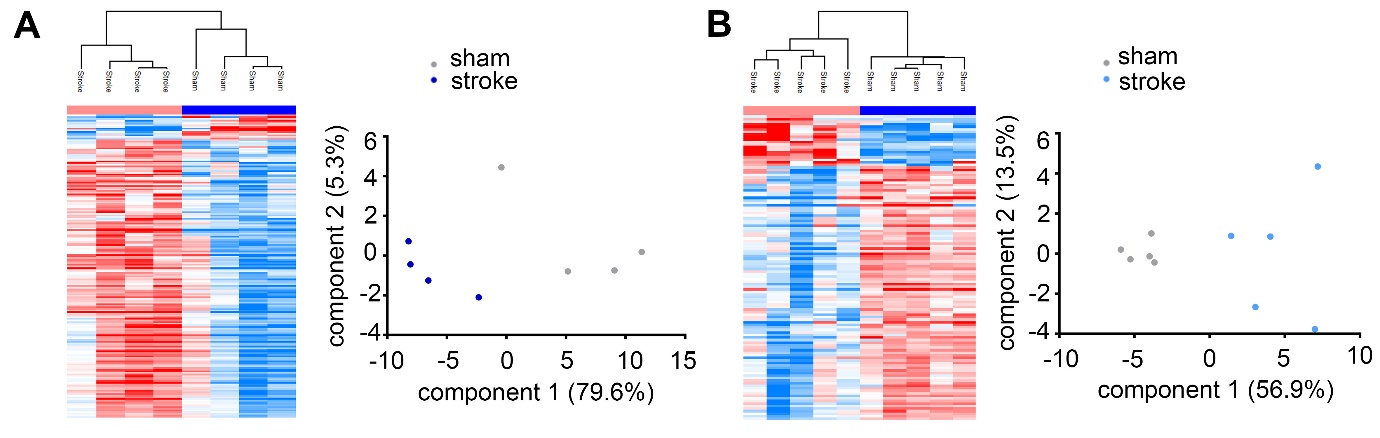
**

**Suppl. Fig. 1. Heat maps and principal component analysis (PCA) of the synaptosome proteomic analysis.** Heat maps and scatter plot visualization of the first two principal components in linear PCA, based on all Student’s *t*-test significant (*p* < 0.05) proteins between sham and stroke at different time points; (A) 24 h and (B) 4 d.

**Suppl. Fig. 2**


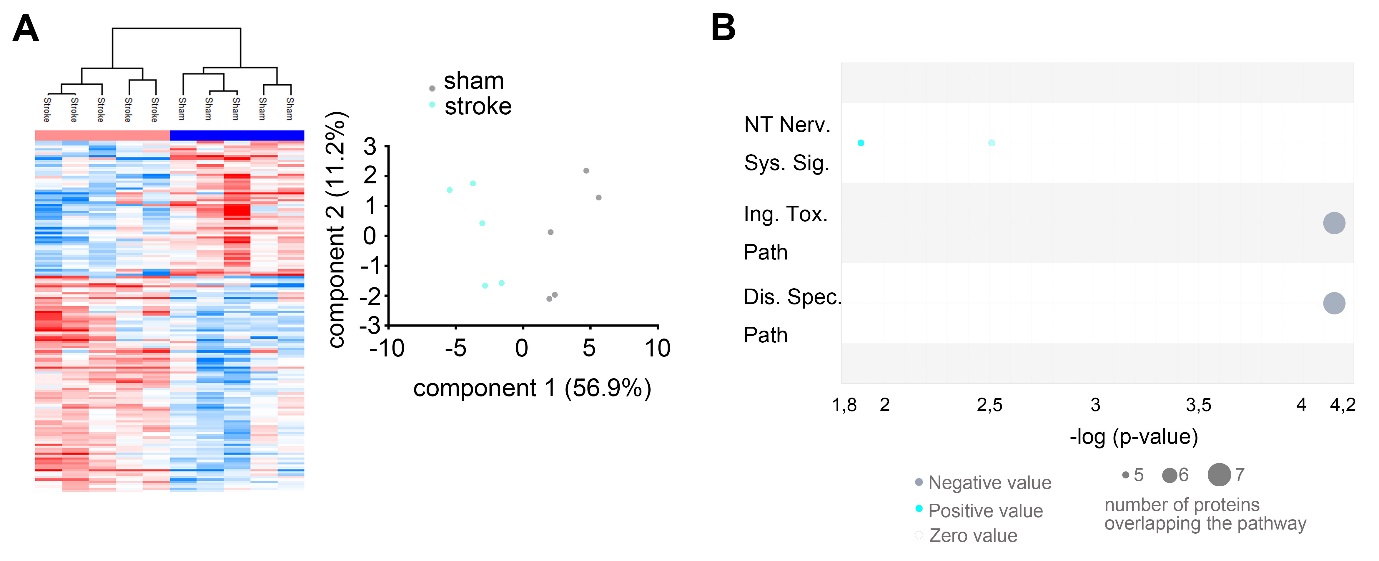


**Suppl. Fig. 2. Heat maps, volcano plots and Ingenuity Pathways Analysis (IPA) 7 days after tMCAO.** (A) Heat map and scatter plot visualization of the first two principal components in linear PCA, based on all Student’s *t*-test significant (*p* < 0.05) proteins between sham and stroke at 7 days after tMCAO. (B) IPA based on proteins quantified 7 d after stroke. A right-tailed Fisher’s Exact Test was used to calculate a *p*-value determining the probability that the association between the genes in the dataset and the canonical pathway is explained by chance alone (*p*-value < 0.05). A z-score was calculated to indicate the likelihood of activation or inhibition of that pathway.

**Suppl. Fig. 3**

**
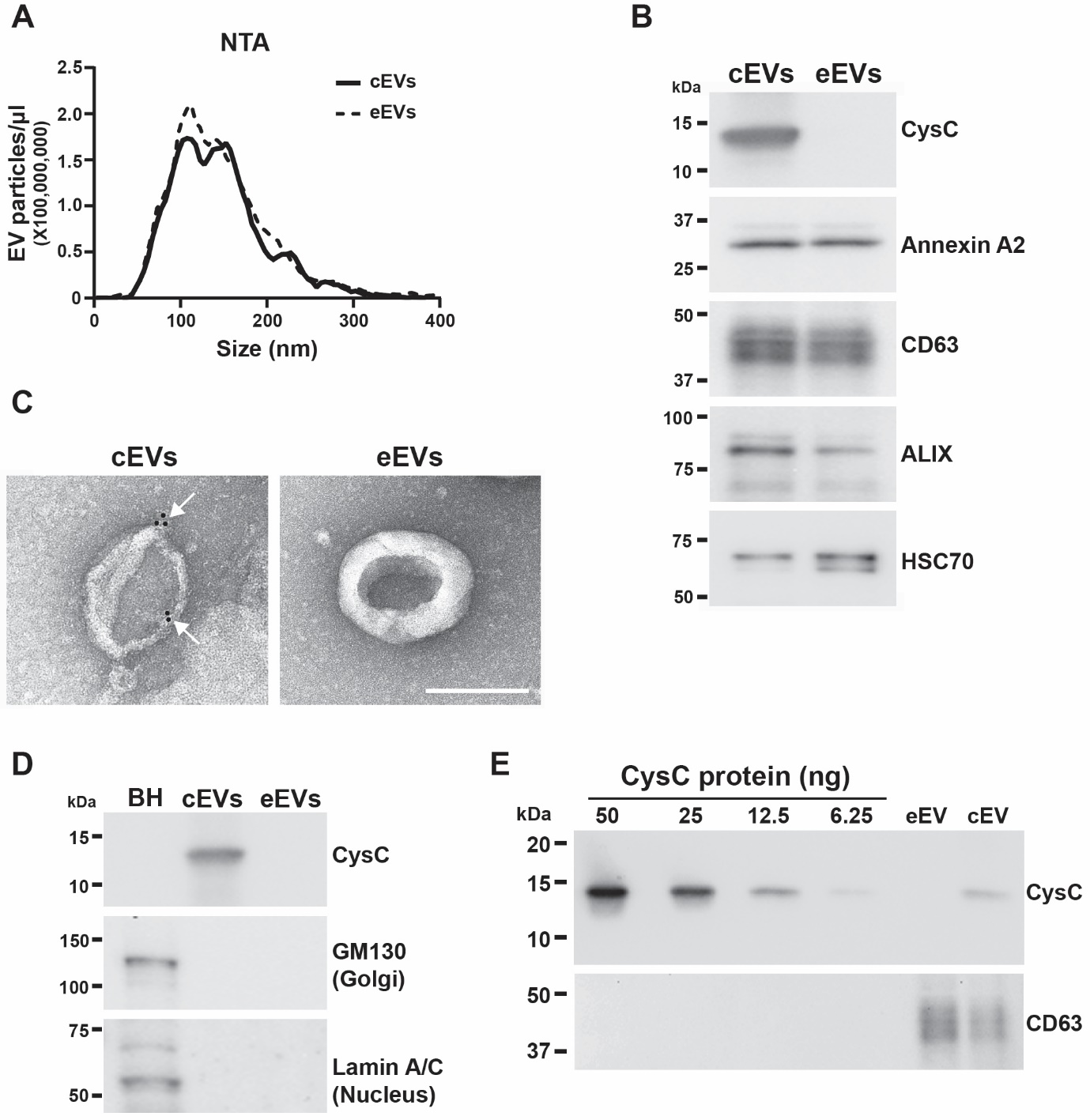
**

**Suppl. Fig. 3. Characterization of BDEVs loaded with CysC.** (A) Representative nanoparticle tracking analysis (NTA) of BDEVs loaded with or without CysC (cEVs and eEVs, respectively) showing no overt differences in size and number of EVs. (B) Representative western blot analyses showing a microvesicle marker (Annexin A2) and exosomal markers (CD63, ALIX, and HSC70) in both cEVs and eEVs. (C) Representative immunoelectron microscopy micrographs (using an anti-CysC antibody) show gold particles bound to the cEVs membrane (arrows), but not to the eEVs, indicating presence of CysC on/in cEVs only. Scale bar: 100 nm. (D) Representative western blot analyses showing CysC loaded into cEVs but not in eEVs and lack of intracellular proteins (GM130 and Lamin A/C present in brain homogenates (BH)) in cEVs and eEVs, confirming the purity of BDEVs. € Western blot analysis shows a quantification of cEVs, which displays the loading of 1.28 ng of CysC in 1 µL of EV suspension (4.5 × 10^9^ EV particles).

**Suppl. Fig. 4.**


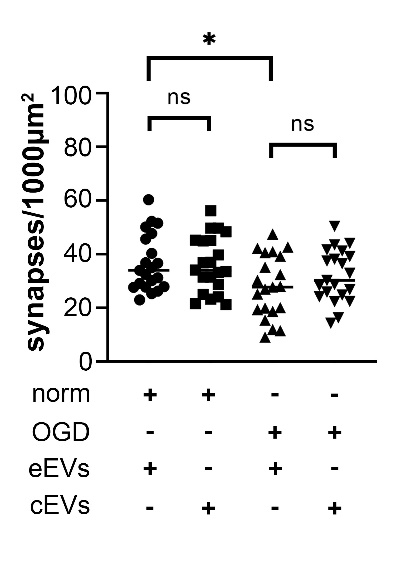


**Suppl. Fig. 4. Treatment with 14 µL of BDEVs loaded with CysC shows no effect on synapses after OGD treatment.** Graph showing the quantification of synapses per mm^2^ under normoxic and OGD conditions after treatment with 14 µL of empty BDEVs (eEVs) or 14 µL of CysC-loaded EVs (cEVs). The quantification shows no significant differences between the treatments in normoxic or under OGD conditions (n=1).

**Suppl. Fig. 5**


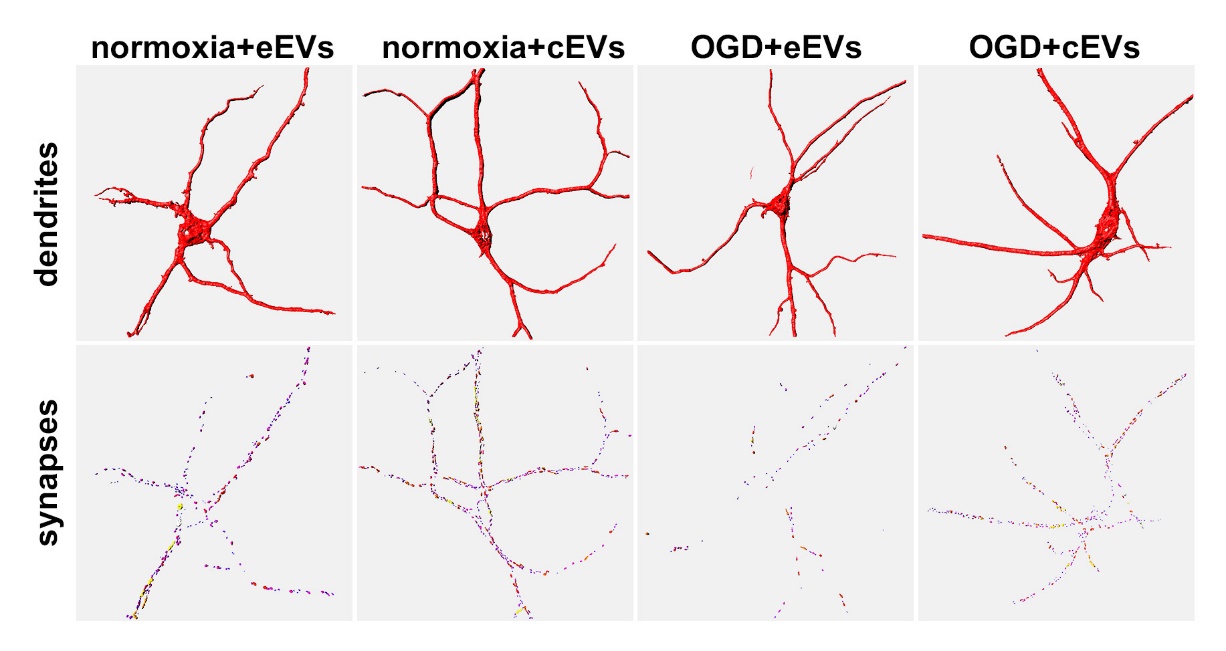


**Suppl. Fig. 5. Representative 3D reconstruction of primary neurons subjected or not to OGD and treated either with eEVs or cEVs.** Dendrites were labeled with MAP2 and synapses with Synapsin 1. The scale bar is 10 µm.

**Suppl. Fig. 6**


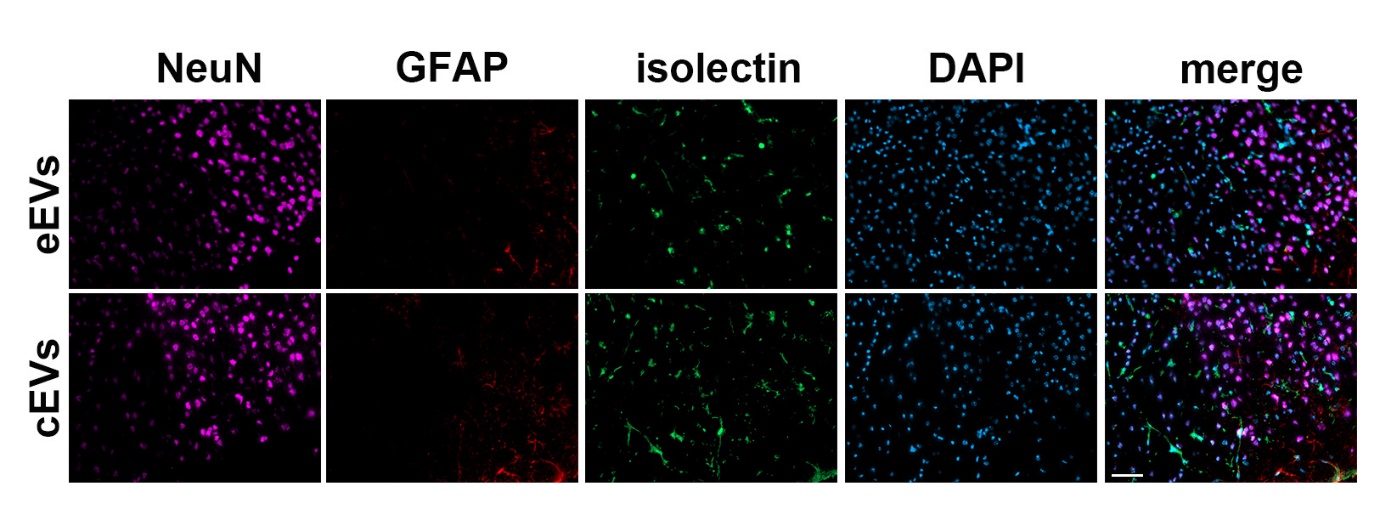


**Suppl. Fig. 6. Microglia and astrocytes show no differences in their immunoreactive pattern between animals treated with eEVs and cEVs.** Representative immunofluorescence pictures of murine brain slices observed under the Apotome microscope (20×). NeuN (violet) labeled neuronal bodies, whereas the microglia were stained with isolectin B4 (green); astrocytes were labeled with GFAP (red), and nuclei were stained with DAPI. No major differences were observed between both groups. +eEVs n=6, +cEVs n=5. The scale bar is 50 µm.

**Suppl. Fig. 7**

**
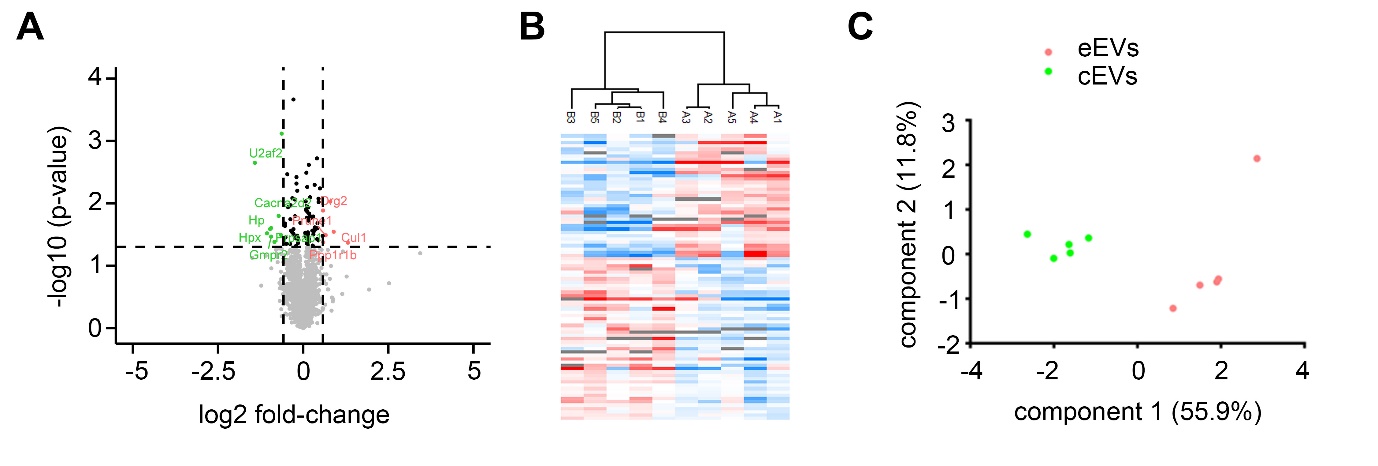
**

**Suppl. Fig. 7. Proteomic analysis of brains of mice subjected to tMCAO and treated with either eEVs or cEVs.** (A) Volcano plot of the -log10 (*p*-value) against the log2FC difference for *t*-testing mass spectrometry results between tMCAO mice treated either with eEVs or with cEVs. (B) Heat map showing differential clustering between tMCAO samples treated with eEVs (red dots) and tMCAO samples treated with cEVs (green dots). (C) Scatter plot visualization of the two main components in supervised PCA, performed based on all Student’s *t*-test significant proteins between tMCAO mouse brains treated either with eEVs or cEVs (n=5 per group).

**SUPPL. TABLES**

**Suppl. Table 1.­ Proteomic data analysis.** List of up and downregulated proteins found after mass spectrometry analysis for each experiment: synaptosomes isolated from shams vs stroke at 24h, 4d and 7d and the comparison of brain homogenates from mice injected with eEVs with mice injected with cEVs.
